# Glycosaminoglycans from a Sea Snake (*Lapemis curtus*): Extraction, Structural Characterization and Antioxidant Activity

**DOI:** 10.3390/md16050170

**Published:** 2018-05-18

**Authors:** Mingyue Bai, Wenwei Han, Xia Zhao, Qingchi Wang, Yanyun Gao, Shiming Deng

**Affiliations:** 1 Key Laboratory of Marine Drugs, Ministry of Education, Shandong Provincial Key laboratory of Glycoscience and Glycoengineering, School of Medicine and Pharmacy, Ocean University of China, Qingdao 266003, China; baimyue@126.com (M.B.); hwwouc@163.com (W.H.); gaoyanyun1991@126.com (Y.G.); 2 Laboratory for Marine Drugs and Bioproducts of Qingdao National Laboratory for Marine Science and Technology, Qingdao 266237, China; 3 Marine Biomedical Research Institute of Qingdao, Qingdao 266071, China; wangqingchi@163.com; 4 Ocean College of Hainan University, Haikou 570228, China; dsm701@126.com

**Keywords:** *Lapemis curtus*, glycosaminoglycans, chondroitin sulfate, dermatan sulfate, structural characterization, antioxidant activities

## Abstract

Sea snakes have wide application prospects in medicine, health food and other fields. Several novel polysaccharides were successfully obtained from the skin and the meat of a sea snake (*Lapemis curtus*). The structures of polysaccharides LSP3 and LMP3, which were extracted and purified from *Lapemis curtus*, were determined to be new and highly heterogenic glycosaminoglycans (GAGs) by means of FT-IR, ESI-MS/MS and NMR. LSP3 is a hybrid dermatan sulfate (DS) and composed of 48% 4-sulfated disaccharides (Di4S), 42% 6-sulfated disaccharides (Di6S) and 5% disulfated disaccharides (Di2,6S), while LMP3 is a hybrid chondroitin sulfate (CS) and composed of 70% Di4S, 20% Di6S, and 8% Di2,6S. More importantly, LSP3 and LMP3 showed a strong scavenging ability of 1,1-diphenyl-2-picrylhydrazyl (DPPH) radicals, iron (Fe^2+^) chelating activity and total antioxidant capacity in vitro, especially LSP3, with high contents of uronic acid and sulfate, which possessed a higher scavenging ability of DPPH radicals than other fractions. These data suggested that the sea snake polysaccharides could be promising candidates for natural antioxidant ingredients.

## 1. Introduction

Glycosaminoglycans (GAGs) are polyanionic polysaccharides composed of amino sugar and uronic acid, which are widely distributed in animals. According to the difference of disaccharide composition and glycosidic bond, GAGs are generally divided into four groups, namely, chondroitin sulfate and dermatan sulfate (CS/DS), keratan sulfate (KS), heparin and heparan sulfate (HP/HS), and hyaluronic acid (HA) [1]. CS and DS have been confirmed to be closely related to inflammation, immune response, cardiovascular disease, tumorigenesis, infection, wound repair and fibrosis, and especially iduronic acid (IdoA), which influences multiple cellular properties [2].

Antioxidant activity is a focus of intensive scientific investigations because of the ever-increasing demand of food and pharmaceutical industries to develop natural antioxidant compounds [3]. The process of oxidative stress plays a major role in the development of chronic and degenerative illness, such as cancer, autoimmune disorders, aging, cataract, rheumatoid arthritis, as well as cardiovascular and neurodegenerative diseases [4]. The human body can counteract oxidative stress by producing antioxidants to protect cells from oxidation. Among these antioxidants, CS and DS have increasingly attracted interest of many research groups [5].

**Figure 1:**
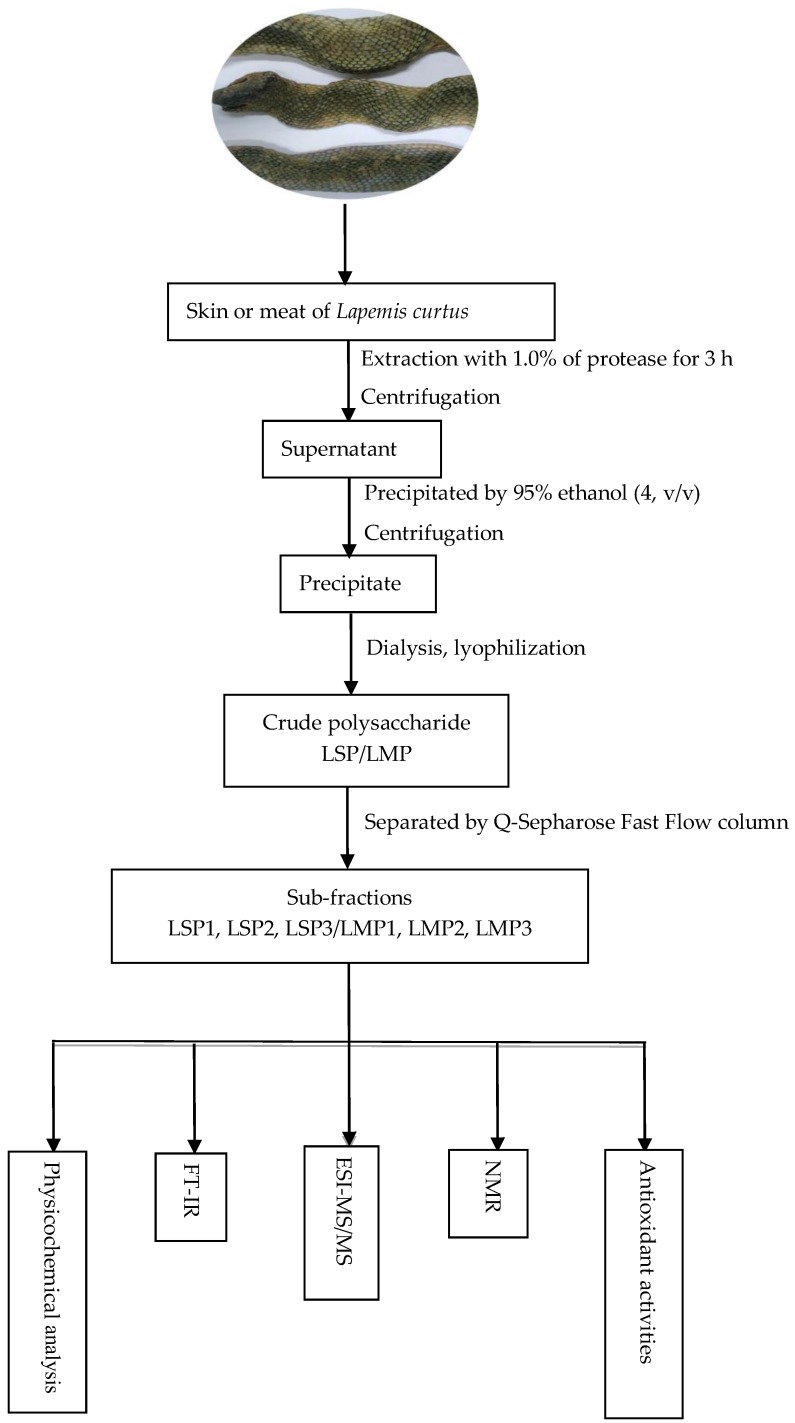
Experimental flowchart of *Lapemis curtus* polysacchloarides.

Sea snakes are the largest group of marine reptiles that inhabit the tropical and subtropical waters of the Indian and Pacific Oceans [6]. Sea snakes have wide application prospects in medicine, health food and other fields. The oil in the viscera of the Erabu sea snake is used as a functional food in Japan [7]. The current research on sea snakes mainly focuses on the snake venom, which is mainly considered to be used in the treatment of infectious, hematological, inflammatory, cardiovascular, and malignant diseases [8]. To the best of our knowledge, there has been no systematic study of sea snake polysaccharides. In this study, several polysaccharides were extracted and purified from the skin and meat of *Lapemis curtus*, and their physicochemical properties, structures, and antioxidant activities were studied (Figure 1). The aim of this study is to provide a basis for further research and development of sea snake polysaccharides.

## 2. Results and Discussion

### 2.1. Chemical Composition

**Figure 2:**
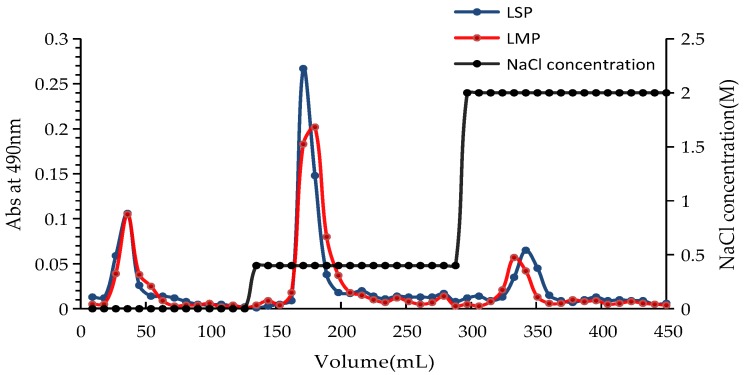
Isolation of the polysaccharides LSP and LMP.

Two crude polysaccharides (LSP and LMP) were obtained by enzymolysis extraction from the skin and the meat of *Lapemis curtus*, respectively. LSP and LMP were further fractionated on a Q Sepharose Fast Flow column (Figure 2), and three sub-fractions of LSP (LSP1, LSP2, and LSP3) and three sub-fractions of LMP (LMP1, LMP2, and LMP3) were obtained. Physicochemical properties of these polysaccharide sub-fractions were analyzed (as shown in Table 1). Non-sulfated polymers were eluted by pure water and a low concentration of NaCl solution (0.4 mol/L), and sulfated polymers were eluted by a high concentration of NaCl solution (2.0 mol/L), both from LSP and LMP. In addition, the fractions eluted by a high concentration of NaCl solution possessed a higher content of uronic acid and a lower content of protein than that of fractions eluted by pure water and a low concentration of NaCl solution. LSP3 displayed a higher content of uronic acid and degree of sulfation than that of LMP3.

**Figure 3:**
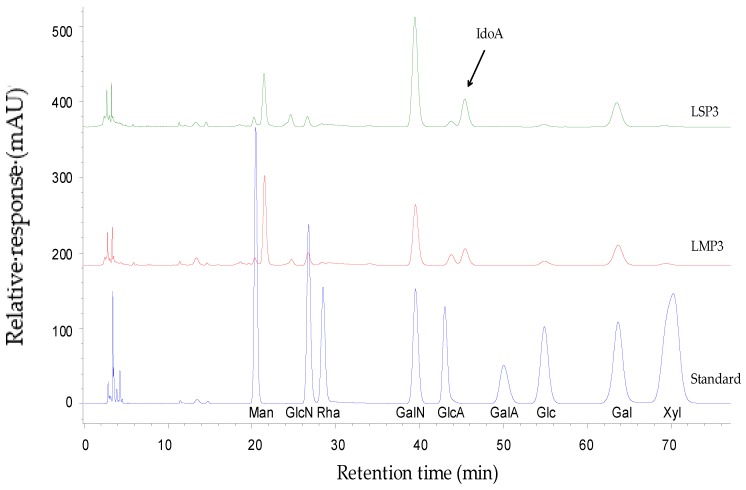
Monosaccharide composition of LSP3 and LMP3. Chromatograms of the acid hydrolysates of LSP3 and LMP3, which were hydrolyzed in 3 mol/L TFA for 3 h at 110 °C to ensure a high response value of IdoA.

Results of monosaccharide composition analysis showed that LSP1 and LMP1, LSP2 and LMP2 were mainly composed of mannose (Man), N-acetylglucosamine (GlcNAc), glucose (Glc) and galactose (Gal), with different molar ratios (Table 1). LSP3 and LMP3 were mainly composed of iduronic acid (IdoA), glucuronic acid (GlcA), N-acetylgalactosamine (GalNAc) and Gal (Figure 3). However, LSP3 contained a high content of IdoA, while LMP3 contained a high content of GlcA. Results of disaccharide compositions analysis showed that LSP3 and LMP3 contained mainly ΔDi6S and ΔDi4S at a molar ratio of 1:1.1 and 1:3.5, respectively (Table 1), that is to say, LSP3 possessed a higher content of ΔDi6S and a lower content of ΔDi4S than that of LMP3. In addition, disulfated disaccharides (ΔDi2,6S) was detected in LSP3 and LMP3, and the contents of ΔDi2,6S were 4.9% and 8.0%, respectively.

### 2.2. FT-IR Spectroscopy of LSP3 and LMP3

**Figure 4:**
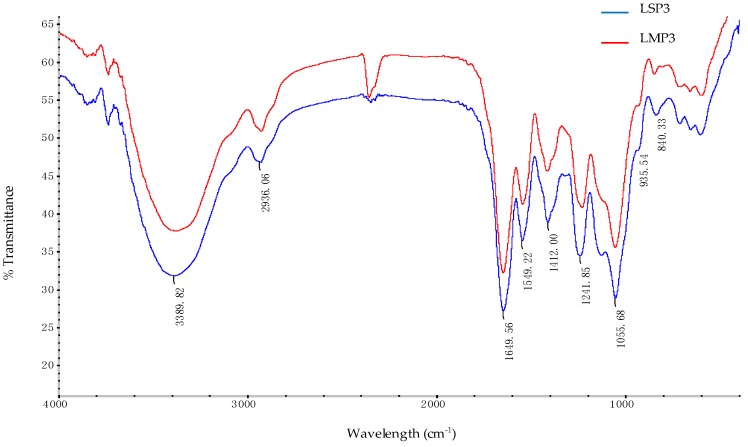
The IR spectra of LSP3 and LMP3.

The FT-IR spectra of LSP3 and LMP3 were shown in Figure 4. The broad and intense absorption at 3389 cm^−1^ was attributed to the O–H stretching vibration. The characteristic C–H stretching vibration of sugar ring was at 2936 cm^−1^. The signal at 1649 cm^−1^ was attributed to the H–O–H vibration. The peak at 1412 cm^−1^ was assigned to the O–H in-plane bending vibration. The signal at 1055 cm^−1^ was related to the C–O–C skeletal vibration. The signals at 1241 cm^−1^ and 840 cm^−1^ were attributed to the stretching vibrations of S=O and C–O–S [9,10], respectively. The peak at 1549 cm^−1^ was assigned to the N–H variable angle vibration. Therefore, it was concluded that LSP3 and LMP3 contain sulfate groups and carboxyl groups, which are in accordance with the structural features of GAGs.

### 2.3. ESI-MS Analysis of LSP3 and LMP3

**Figure 5:**
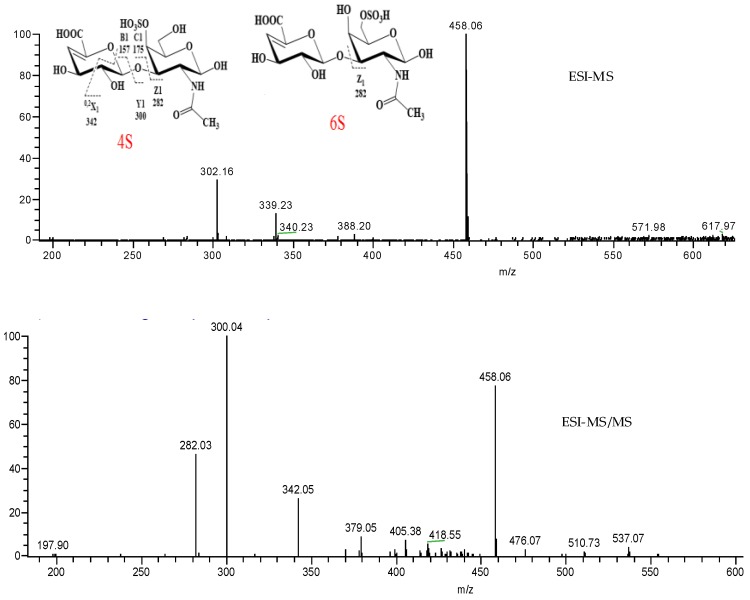
The ESI-MS and MS/MS spectra of LSP3. The green lines are arrow marks from the software system.

ESI-MS is a soft ionization technique commonly coupled with liquid chromatography for identification of oligosaccharides. As shown in Figure 5, the main deprotonated ion [M − H]^−^ of LSP3 was produced at *m*/*z* 458.06 in negative ESI-MS mode, which is in accordance with the structure of ΔHexA-GalNAc, with one sulfate group digested by chondroitinase ABC afterwards. In order to identify the substitution and the linkage of this fraction, the singly charged molecule ion [M − H]^−^ *m*/*z* 458.06 was further selected as the precursor ion for ESI-MS/MS analysis. The ion *m*/*z* 282.03, produced from ΔDi6S cleavage, was assigned as Z_1_ from the reducing terminal. The ions *m*/*z* 342.05, *m*/*z* 300.04 and *m*/*z* 282.03, produced from ΔDi4S cleavage, were assigned as ^0,2^X_1_, Y_1_ and Z_1_ from the reducing terminal [11], respectively. The results showed that LSP3 contains mainly ΔDi6S and ΔDi4S, which are in close agreement with the analysis of disaccharide compositions. The ESI-MS spectrum of LMP3 is essentially identical to that of LSP3.

### 2.4. NMR Spectroscopy Analysis of LSP3 and LMP3

**Figure 6:**
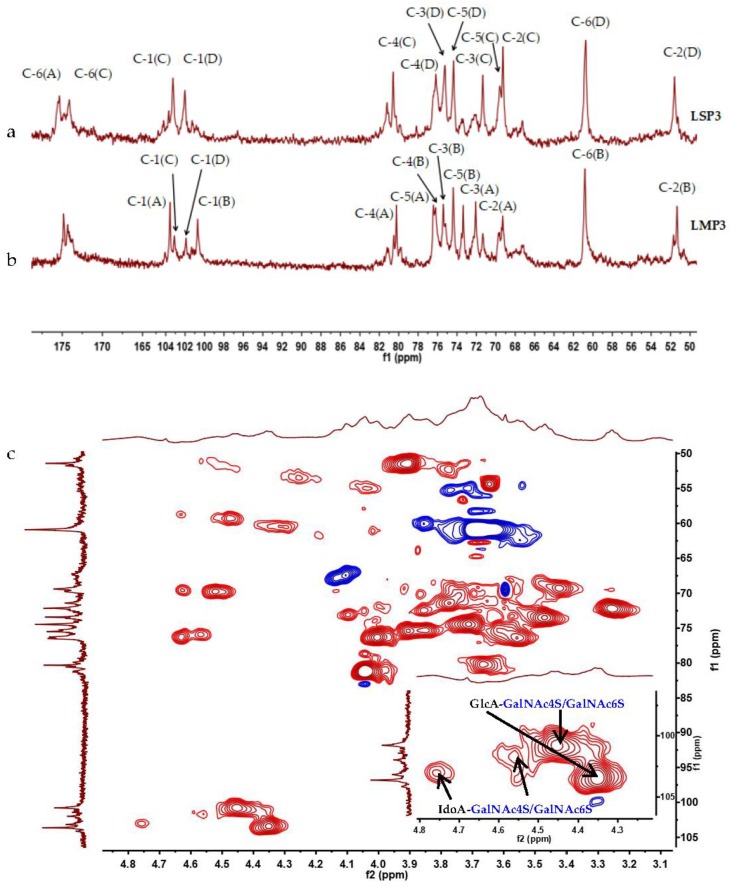
The ^13^C-NMR spectra of LSP3 (**a**) and LMP3 (**b**), and the HSQC spectrum of LMP3 (**c**).

The structures of LSP3 and LMP3 were further elucidated by means of ^1^H-NMR and ^13^C-NMR (Figure 6) spectroscopy. Take LMP3, for example: four major anomeric carbon signals at δ 103.35 ppm (**A**), 101.08 ppm (**B**), 103.13 ppm (**C**) and 101.68 ppm (**D**) were observed in the ^13^C-NMR spectrum (Figure 6b), and four protons at δ 4.35 ppm (**A**), 4.44 ppm (**B**), 4.77 ppm (**C**) and 4.56 ppm (**D**) were observed accordingly in the ^1^H-NMR spectrum. The presence of CS and DS units in the structures of LMP3 were confirmed by two series of signals related to uronic acid (**A**, **C**) and GalNAc (**B**, **D**) residues, as the previous report indicated [1]. A series of 2D-NMR (NOESY, TOCSY, COSY, HSQC, HMBC) experiments allowed almost complete assignment of GlcA-GalNAc4S, GlcA-GalNAc6S, IdoA-GalNAc4S and IdoA-GalNAc6S disaccharide fragments of LSP3 and LMP3 (as listed in Table 2) [1,12,13]. The signals of **B**(H1)-**B**(C1) (4.44 ppm/101.08 ppm) and **D**(H1)-**D**(C1) (4.56 ppm/101.68 ppm) in the HSQC spectrum of LMP3 (Figure 6c) were attributed to GlcA-GalNAc4S and IdoA-GalNAc4S disaccharides of LMP3, due to the different chemical environment for GalNAc sugar ring. By calculating the signal intensities of C-1(**A**)/C-1(**C**), it was found that LSP3 and LMP3 contained GlcA and IdoA, with a molar ratio of 2.9:7.1 and 7.0:3.0, respectively. The structures of LSP3 and LMP3 were determined as CS/DS chains. LSP3 is a DS-rich GAG and LMP3 is a CS-rich GAG.

**Figure 7:**
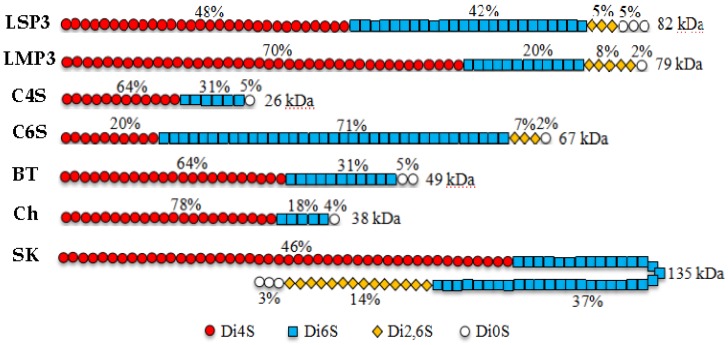
Schematic representation of the structure of LSP3, LMP3 and CS extracted from the cartilage of bovine trachea (BT), chicken sternum (Ch) and skate (Sk). Two commercial preparations were also used: C4S, from bovine trachea, and C6S, from shark cartilage [15].

GAGs in marine animals are different to those of terrestrial organisms, mainly in terms of molecular weight and sulfation pattern. CS extracted from squid possesses antiviral and anti-metastatic activities. DS from sea squirts and hybrids CS/DS from sharks can promote the outgrowth of neurite, and are useful for nerve regeneration [14]. CS and DS are complex molecules with potential impacts on many biological systems, and it is important to consider the sulfation pattern and the size of the molecules to better understand the structure/function relationships of CS/DS [15]. The structural schematic representations of LSP3 and LMP3 were shown in Figure 7, based on the analysis of disaccharide compositions, ESI-MS/MS and NMR. The structures of LSP3 and LMP3 are different in sulfation patterns and molecule sizes compared with the structures of CS extracted from the cartilage of different animal species [15]. LSP3 and LMP3 possessed higher molecular weight than the C4S, bovine trachea (BT) and chicken sternum (Ch) of terrestrial organisms. Especially, LSP3 and LMP3 contain significant amounts of disulfated disaccharides (Di2,6S), which is only found in ocean animals, like sharks and skates. Therefore, LSP3 and LMP3 are new structure types of GAGs, which might display different functions.

### 2.5. Antioxidant Activity of Fractions

**Figure 8:**
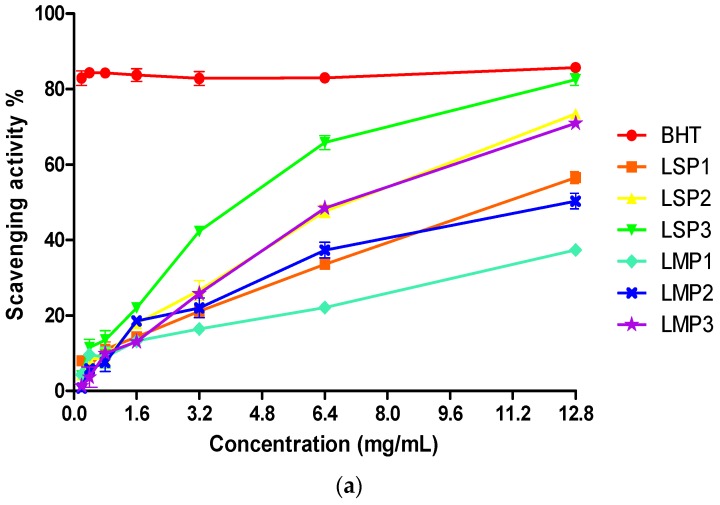

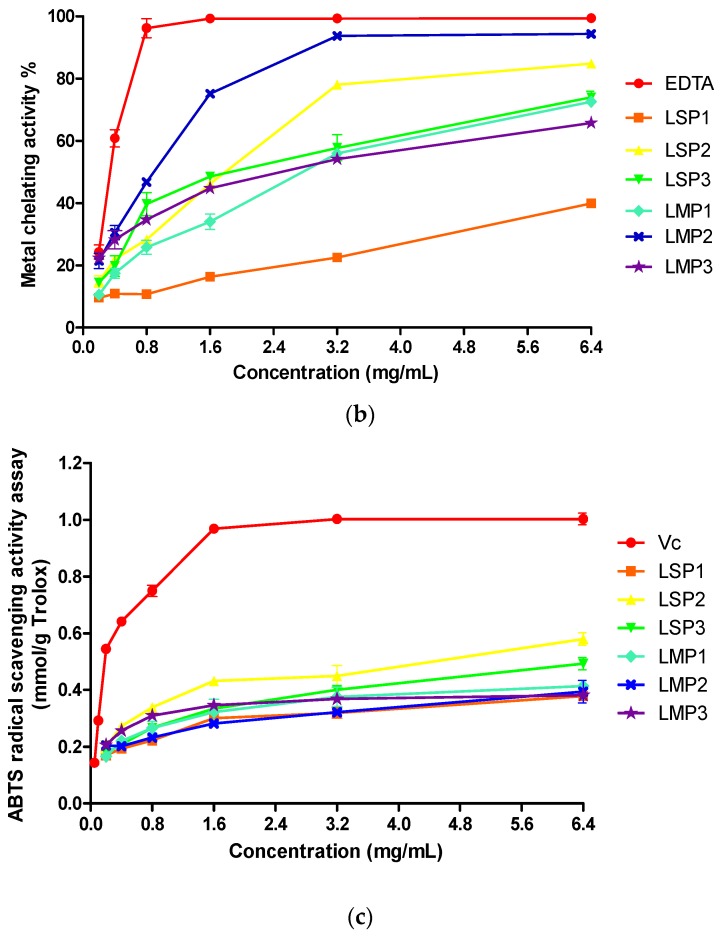
Antioxidant properties of polysaccharide sub-fractions from *Lapemis curtus*. (**a**) DPPH radical scavenging activities, (**b**) iron chelating effect, (**c**) 2,2′-azino-bis(3-ethylbenzothiazoline-6-sulfonic acid) (ABTS) radical scavenging activities of different samples. LSP1, LSP2 and LSP3, extracted from the skin of *Lapemis curtus.* LMP1, LMP2 and LMP3 extracted from the meat of *Lapemis curtus.* BHT, EDTA and Vc were used as positive controls.

DPPH is a useful reagent to evaluate the free radical scavenging ability of hydrogen-donating antioxidants, which can transfer hydrogen atoms or electrons to DPPH radicals. Iron is the most abundant transition metal in biological systems and plays critical roles in redox systems. ABTS [2,2′-azino-bis(3-ethylbenzothiazoline-6-sulfonic acid)] radical cation scavenging assay is an excellent tool for determining the antioxidant activity of hydrogen-donating antioxidants and chain-breaking antioxidants. The results showed that the DPPH radicals’ scavenging ability, ferrous chelating power and total antioxidant capacity of each *Lapemis curtus* polysaccharides was concentration-dependent (Figure 8), and all the fractions exhibited significant antioxidant activity, especially for LSP3.

The fractions LSP1, LSP2, LSP3 from the skin of *Lapemis curtus* exhibited stronger scavenging activity than that of fractions LMP1, LMP2, LMP3 from the meat of *Lapemis curtus*, which eluted at the same concentration of NaCl on a Q-Sepharose Fast Flow column. Especially, the scavenging ability of LSP3 on DPPH radicals was up to 65.8% at 6.4 mg/mL (Figure 8a), which was similar to that of Butylated hydroxytoluene (BHT). The fractions LMP1, LMP2 from the meat of *Lapemis curtus* exhibited higher chelating activities than that of fractions LSP1, LSP2 from the skin of *Lapemis curtus*. LMP2 exhibited the most effective chelating activity fraction, followed by LSP2 and LSP3. In addition, the chelating activity of LMP2 was up to 93.7% at 3.2 mg/mL (Figure 8b), which was similar to that of Ethylene diamine tetraacetic acid (EDTA). The highest total antioxidant capacities were recorded in LSP2 and LSP3 (Figure 8c), with 57.8% and 49.2% of total antioxidant capacities at 6.4 mg/mL, respectively.

The results showed that DPPH and Fe^2+^ were more sensitive to *Lapemis curtus* polysaccharides than ABTS. LSP3 and LMP3 with high DS/CS content possessed a strong scavenging ability of DPPH radicals, iron (Fe^2+^) chelating activity and total antioxidant capacity. In addition, LSP3 possessed stronger antioxidant activities than that of LMP3, which may be related to its high content of uronic acid and degree of sulfation. LSP2 possessed a strong scavenging ability of DPPH radicals, iron (Fe^2+^) chelating activity and total antioxidant capacity, which may be related to its high contents of GlcNAc [16] and proteins. The polysaccharides from *Lapemis curtus* showed great potential for future human health applications.

## 3. Materials and Methods

### 3.1. Materials and Reagents

*Lapemis curtus* was provided by the Ocean College of Hainan University (Haikou, Hainan Province, China). Chondroitinase ABC was provided by the Marine Biomedical Research Institute of Qingdao (Qingdao, Shandong Province, China). Neutral protease of *Bacillus subtilis* was purchased from Novozymes (Copenhagen, Denmark), papain was purchased from AppliChem (Darmstadt, Germany), and trypsin was purchased from Amresco (Washington, USA). Monosaccharide standards of glucose (Glc), galactose (Gal), glucosamine (GlcN), xylose (Xyl), arabinose (Ara), mannose (Man), rhamnose (Rha), fucose (Fuc), galactosamine (GalN), glucuronic acid (GlcA), galacturonic acid (GalA), and unsaturated disaccharides standards of ΔUA-GalNAc (ΔDi0S), ΔUA-GalNAc6S (ΔDi6S), ΔUA-GalNAc4S (ΔDi4S), ΔUA2S-GalNAc6S (ΔDi2,6S), ΔUA4S-GalNAc6S (ΔDi4,6S), ΔUA2S-GalNAc4S (ΔDi2,4S), ΔUA2S-GalNAc4S6S (ΔDi2,4,6S), were purchased from Sigma-Aldrich (St. Louis, MO, USA). HPLC-grade acetonitrile was purchased from Merck KGaA (Darmstadt, Germany). All of the other chemicals and solvents used were of analytical grade, unless otherwise specified.

### 3.2. Extraction and Purification

Polysaccharides were extracted from the skin and the meat of *Lapemis curtus*. The skin of *Lapemis curtus* was minced and digested with 1.0% of papain and trypsin at a ratio of 1:2 (*v*/*v*) at 50 °C for 3 h. The digested mixture was centrifuged, and the supernatant was precipitated by four volumes of ethanol (95%). The precipitate was resuspended and dialyzed against water using a 1 kDa MWCO (molecular weight cut off) dialysis tube, and then freeze-dried to obtain a crude polysaccharide from the skin (named LSP). The meat of *Lapemis curtus* was minced and digested with 1.0% of neutral protease of *Bacillus subtilis* at 55 °C for 3 h, and a crude polysaccharide from meat (named LMP) was obtained after a similar process as that of LSP. LSP and LMP were further fractionated on a Q-Sepharose Fast Flow column and eluted with a step-wise gradient of 0, 0.4 and 2.0 mol/L NaCl solution to obtain LSP1, LSP2, LSP3, and LMP1, LMP2, LMP3, respectively. Finally, the purified components were pooled, dialyzed and lyophilized.

### 3.3. Chemical Analysis and Molecular Weight Analysis

Total uronic acid content was determined by a colorimetrical method [17] using glucuronic acid as a standard. Protein content was measured by a Bicinchoninic Acid Protein Assay Kit (BCA kit) (Sigma-Aldrich, 3050 Spruce Street, St Louis, MO 63103, USA) [18] using bovine serum albumin as a standard. Sulfate content was assayed using an ion chromatography method [19]. Molecular weight (Mw) was determined by a high-performance liquid chromatography, coupled with a refractive index detector (Agilent Technologies, Wilmington, DE, USA), with a column of TSKgel G3000PW_XL_ (TOSOH, Tokyo, Japan). Aqueous Na_2_SO_4_ solution (0.1 mol/L) was used as the mobile phase and the flow rate was 0.5 mL/min. The temperature of the column was maintained at 35 °C. Dextrans were used as standards to calibrate the column [20].

### 3.4. Composition Analysis

Monosaccharide composition was determined using a 1-phenyl-3-methyl-5-pyrazolone (PMP) pre-column derivatization HPLC method [21]. The PMP-labeled carbohydrates were separated by a BDS-C_18_ column (4.6 mm × 250 mm, 5 µm, Hypersil, Waltham, MA, USA) with 0.1 mol/L phosphate buffer (pH 6.0) and acetonitrile at a ratio of 84:16 (*v*/*v*, %) as a mobile phase at a flow rate of 1.0 mL/min. Disaccharide composition analysis was performed by enzymatic degradation and chromatographic separation on a Zorbax SAX column (9.4 mm × 250 mm, 4.6 µm). A gradient elution was performed using pure water and 2 mol/L NaCl solution (pH = 3.5) as a mobile phase at a flow rate of 1.0 mL/min. The identification and quantitation of each unsaturated disaccharide was performed by comparing with standard disaccharides [22].

### 3.5. Fourier Transform Infrared (FT-IR) Spectroscopy Analysis

The FT-IR spectra of *Lapemis curtus* polysaccharides were recorded on a Nexus 470 FT-IR spectrophotometer (Nicolet, Pleasanton, CA, USA) in KBr pellets over a wavelength range of 400 cm^−1^–4000 cm^−1^.

### 3.6. Electrospray Mass Spectroscopy (ESI-MS) Analysis

Negative-ion ESI-MS/MS analysis was carried out on a Micromass LTQ-Orbitrap XL instrument (Thermo Fisher Scientific, Waltham, MA, USA). Nitrogen was used as sheath gas at a flow rate of 8 arb. The capillary temperature was 275 °C. The spray voltage, capillary voltage, and tube lens voltage were 3 KV, 43 V, and 80 V, respectively. The mobile phase was acetonitrile/H_2_O (1:1, *v*/*v*) at a flow rate of 10 μL/min. All of the samples were dissolved in mobile phase before injection [23].

### 3.7. NMR Spectroscopy Analysis

The lyophilized polysaccharides (20–30 mg) were co-evaporated with D_2_O (99.96%) three times to remove the exchangeable protons and then finally dissolved in 500 μL D_2_O. Deuterated acetone was used as an internal standard (2.08 ppm for ^1^H-NMR and 29.34 ppm for ^13^C-NMR). ^1^H-NMR, ^13^C-NMR, ^1^H-^1^H COSY, HSQC, HMBC, TOCSY and NOESY experiments were recorded at 298 K on an Agilent DD2-500 spectrometer (Agilent Technologies, Wilmington, DE, USA) [24].

### 3.8. Determination of Antioxidant Activity

#### 3.8.1. DPPH Free Radical Scavenging Activity

The scavenging ability of *Lapemis curtus* polysaccharides on DPPH radicals was measured as previously described [25]. Briefly, 100 μL of sample solution at different concentrations was added to 400 μL of 0.004% ethanol solution of DPPH. Absorbance at 517 nm was measured after 30 min. BHT was used as a positive control. The scavenging ability was calculated as follows:

Scavenging ability (%) = (1 − *A*_sample_/*A*_control_) × 100.

where *A*_control_ is the absorbance of control without test samples, and *A*_sample_ is the absorbance in the presence of test samples. The test was carried out in triplicate.

#### 3.8.2. Iron (Fe^2+^) Chelating Activity

The iron chelating effect of *Lapemis curtus* polysaccharides was tested as previously described [26]. Briefly, 50 μL of sample solution at different concentrations was mixed with 25 μL of 0.5 mmol/L FeCl_2_ and 225 μL of methanol solution. The mixtures were incubated at room temperature for 5 min and the reaction was initiated by the addition of 100 μL of 5 mmol/L ferrozine solution. The mixtures were then vigorously shaken and remained at room temperature for 10 min. EDTA was used as a positive control. The absorbance of solution was measured at 562 nm, and the chelating activity (%) was calculated as follows:

Metal chelating activity (%) = (1 − *A*_sample_/*A*_control_) × 100

where *A*_control_ is the absorbance of control without test samples, and *A*_sample_ is the absorbance of test samples. The test was carried out in triplicate.

#### 3.8.3. Total Antioxidant Capacity Assay Kit with ABTS Method

The total antioxidant capacity of *Lapemis curtus* polysaccharides was measured by the ABTS method, as previously described [27]. The working solution was prepared by mixing ABTS solution and oxidant solution in equal quantity and remained in the dark at room temperature for 16 h. Next, 10 μL of sample solution at different concentrations were mixed with 200 μL of diluted ABTS solution and then stored at room temperature for 6 min. The absorbance of solution was measured at 734 nm. Vitamin C (Vc) and trolox were used as positive controls. Trolox, a water-soluble analogue of vitamin E, was used as a reference standard to prepare a calibration curve at a concentration range of 0.05–1.6 mmol/L. Results were expressed as mmol/g Trolox equivalent antioxidant capacity (TEAC). The test was carried out in triplicate.

## 4. Conclusions

Two crude polysaccharides were extracted from the skin and the meat of *Lapemis curtus* (LSP and LMP), and further purified to obtain polysaccharide sub-fractions of LSP1, LSP2, LSP3 and LMP1, LMP2, LMP3, respectively. The structures of LSP3 and LMP3 were determined as a new hybrid CS/DS by means of IR, ESI-MS, NMR and composition analysis. LSP3 is a DS enriched GAG and LMP3 is a CS enriched GAG. The polysaccharides extracted from *Lapemis curtus* exhibited significant antioxidant activities. Especially, LSP3 possessed a strong scavenging ability of DPPH radicals, iron (Fe^2+^) chelating activity and total antioxidant capacity, and this may be related to its high contents of uronic acid and sulfate. Our data suggested that the polysaccharides from *Lapemis curtus* could be promising candidates for natural antioxidant ingredients.

## Figures and Tables

**Table 1:** The chemical compositions of each fraction from *Lapemis curtus*. The total mole number of unsaturated disaccharides of each sample was taken as 100% in disaccharide compositions. “-” represents not detected.

Composition	Polysaccharides from Skin			Polysaccharides from Meat		
	LSP1	LSP2	LSP3	LMP1	LMP2	LMP3
Uronic acid (%)	0.5	1.8	25.3	1.1	1.9	15.2
Total proteins (%)	48.3	46.0	19.2	42.4	63.8	12.3
Sulfated groups (%)	0.7	0.4	11.2	0.5	0.4	10.1
Molecular weight (kDa)	3.7	3.1	82.0	2.1	2.7	79.0
Monosaccharide (molar ratio)						
Mannose	5.6	4.7	-	4.8	2.5	-
N-acetyl Glucosamine	12.6	17.2	1.8	18.1	13.8	2.0
Rhamnose	-	-	-	-	-	-
Glucuronic acid	-	1.1	16.2	-	-	12.3
Galacturonic acid	-	-	-	-	-	-
N-acetyl Galactosamine	1.1	1.6	30.1	2.3	1.5	15.9
Glucose	9.4	3.3	1.2	15.4	3.1	2.0
Galactose	22.9	14.6	10.8	19.9	8.8	8.8
Xylose	-	-	1.3	-	-	1.5
Arabinose	-	-	-	1.1	1.6	-
Fucose	1	1	1	1	1	1
Disaccharide (%)						
ΔDi0S			5.0			1.6
ΔDi6S			42.2			20.0
ΔDi4S			47.9			70.5
ΔDi2,6S			4.9			8.0

**Table 2:** The ^1^H-NMR and ^13^C-NMR assignments of LSP3 and LMP3.

Signal/ppm		H1	H2	H3	H4	H5	H6	Residue
		(C1)	(C2)	(C3)	(C4)	(C5)	(C6)	
**A**	→4)-β-GlcA-(1→	4.35	3.27	3.47	3.66	3.57	-	→4)-β-GlcA-(1→3)-β-GalNAc4S-(1→
		(103.35)	(72.31)	(73.46)	(80.18)	(76.42)	(174.27)	
**B**	→3)-β-GalNAc4S-(1→	4.44	3.91	3.90	4.63	3.71	3.69	
		(101.08)	(51.43)	(75.38)	(76.28)	(74.45)	(60.83)	
**C**	→4)-β-IdoA-(1→	4.77	3.42	3.78	3.97	4.60	-	→4)-β-IdoA-(1→3)-β-GalNAc4S-(1→
		(103.13)	(69.33)	(71.46)	(80.53)	(69.72)	(173.62)	
**D**	→3)-β-GalNAc4S-(1→	4.56	3.93	3.84	4.55	3.71	3.69	
		(101.68)	(51.76)	(75.25)	(76.09)	(74.45)	(60.83)	
**A′**	→4)-β-GlcA-(1→	4.40	3.16	3.50	3.59	3.56	-	→4)-β-GlcA-(1→3)-β-GalNAc6S-(1→
		(104.00)	(72.29)	(73.41)	(81.20)	(76.39)	(174.27)	
**B′**	→3)-β-GalNAc6S-(1→	4.42	3.91	3.74	4.00	3.86	4.10	
		(101.27)	(51.41)	(74.50)	(67.71)	(72.46)	(67.51)	
**C′**	→4)-β-IdoA-(1→	4.73	3.50	3.74	ND	ND	-	→4)-β-IdoA-(1→3)-β-GalNAc6S-(1→
		(102.98)	(69.00)	(70.85)	ND	ND	(173.62)	
**D′**	→3)-β-GalNAc6S-(1→	4.50	3.93	3.75	4.03	ND	4.13	
		(101.68)	(51.76)	(80.21)	(68.13)	ND	(67.90)	
